# Neurocognition, cerebellar functions and psychiatric features in spinocerebellar ataxia type 34: a case series

**DOI:** 10.3389/fncom.2025.1710961

**Published:** 2025-12-09

**Authors:** Maurizio Cundari, Lena Kirchhoff, Susanna Vestberg, Danielle van Westen, Sigurd Dobloug, Karin Markenroth Bloch, Markus Nilsson, Linda Wennberg, Boel Hansson, Nikos Priovoulos, Anders Rasmussen, Sorina Gorcenco

**Affiliations:** 1 Department of Experimental Medical Science, Faculty of Medicine, Lund University, Lund, Sweden; 2 Unit of Neurology, Hospital of Helsingborg, Helsingborg, Sweden; 3 Unit of Neuropsychiatry, Hospital of Helsingborg, Helsingborg, Sweden; 4 Department of Psychology, Faculty of Social Science, Lund University, Lund, Sweden; 5 Institution for Clinical Sciences Lund, Lund University, Lund, Sweden; 6 Image and Function, Skane University Hospital, Lund, Sweden; 7 Department for Clinical Sciences Lund, Neurology, Skane University Hospital, Lund University, Lund, Sweden; 8 Lund University Bioimaging Centre, Lund University, Lund, Sweden; 9 Department of Clinical Sciences Lund, Radiology, Lund University, Lund, Sweden; 10 Nuffield Department of Clinical Neurosciences, Oxford University Centre for Integrative Neuroimaging, FMRIB, University of Oxford, Oxford, United Kingdom; 11 Spinoza Centre for Neuroimaging, Royal Netherlands Academy for Arts and Sciences, Amsterdam, Netherlands

**Keywords:** cerebellum, neurocognition, neuropsychology, neurology, neurogenetics, neuroimaging, 7T MRI, spinocerebellar ataxia

## Abstract

**Objective:**

This study primarily aimed to comprehensively characterize the neurological, neuroradiological and neurocognitive profiles, as well psychiatric features of individuals with Spinocerebellar Ataxia Type 34 (SCA34) associated with pathogenic variants in the *ELOVL4* gene. Secondarily, we investigated the relationship between neurocognitive functions and cerebellar morphology in individuals with SCA34 by correlating structural changes to cognitive performance. Given involvement of the cerebellum in SCA34, our findings will contribute to a broader understanding of the role of the cerebellum in cognition.

**Methods:**

Four individuals (52 f, 72 m, 76 m, 76 f) underwent DNA testing using Next-Generation Sequencing and detailed assessment of neurocognitive functions. The test battery evaluated all six cognitive domains: verbal functions, executive functions, attention and processing speed, learning and memory, visuospatial perception and abilities, and social cognition. In addition, cerebellar and motor functions were evaluated using Finger Tapping, Prism Adaptation, and the Motor Speed subtest of the Delis-Kaplan executive function system (D-KEFS). Test results were compared with each individual’s estimated premorbid cognitive level, determined from their highest educational attainment or occupational status prior to disease onset. Psychiatric symptoms related to anxiety, depression, and sleep were reported using clinical scales. The Scale for the Assessment and Rating of Ataxia (SARA) was used to assess ataxia severity. Two individuals and one matched control underwent high-resolution 7T MRI to characterize cerebellar morphology.

**Results:**

Neurocognitive assessments identified cognitive and motor dysfunction across all individuals, including distinct neurocognitive impairments consistent with cerebellar cognitive-affective syndrome (CCAS), along with additional deficits in learning, visual and verbal episodic memory, emotion recognition—a component of social cognition. Anxiety and sleep disturbance, but not depression, were observed in both female participants. High-resolution 7 T MRI revealed structural cerebellar alterations, with moderate to severe bilateral cerebellar atrophy, including the vermis and multiple lobules (Crus II, VIIb, VIIIa, VIIIb, IX), as well as atrophy of the middle and superior cerebellar peduncles, accompanied by mild pontine atrophy. Genetic analyses confirmed the involvement of *ELOVL4*-related disruptions in long-chain fatty acid biosynthesis, offering insight into the molecular underpinnings of cerebellar degeneration in SCA34.

**Conclusion:**

Individuals with SCA34 show cerebellar degeneration accompanied by cognitive, motor, and social-affective impairments consistent with CCAS. Atrophy of the vermis, multiple lobules, and cerebellar peduncles align with these deficits, highlighting the cerebellum’s key role in cognition. ELOVL4-related disruptions in fatty acid biosynthesis provides insight into the molecular basis of SCA34. Together, these findings advance our understanding of how cerebellar pathology contributes to complex neurocognitive and psychiatric symptoms in genetic ataxias.

## Introduction

Spinocerebellar ataxia type 34 (SCA34) is a rare neurodegenerative disorder linked to pathogenic variants in the *ELOVL4* gene (Beaudin et al., 2020). It is associated with progressive neurological impairments, neurocognitive deficits, and psychiatric symptoms (Beaudin et al., 2020; Cundari et al., 2023). SCA34 is distinctive in that it often co-occurs with erythrokeratodermia—brownish-red skin lesions typically found on the legs, forearms, and wrists—resulting from mutations in the *ELOVL4* gene, which plays a key role in the elongation of long-chain fatty acids (Beaudin et al., 2020; Mukherjee et al., 2021). While some aspects of SCA34 have been described, a comprehensive understanding of its neurological features, neurocognitive profiles, psychiatric and morphological is still missing. Further characterization of spinocerebellar ataxias may increase our understanding of the contribution of the cerebellum to cognition and provide insight into how specific cerebellar regions relate to cognitive and affective dysfunctions. Spinocerebellar ataxias are often associated with “*cerebellar cognitive affective syndrome*” (CCAS), which encompasses deficits in executive functions, language, visuospatial organization, and emotional regulation (Hirsch et al., 2025). This syndrome highlights the cognitive and affective implications of cerebellar atrophy.

Historically, the cerebellum has been primarily associated with regulating movement and refining motor skills through mechanisms of error-based learning (Marr, 1969). However, emerging findings highlight the cerebellum’s involvement in higher-order cognitive functions that extend beyond motor precision (Cundari et al., 2023; Manto et al., 2024). A central feature of cerebellar processing involves the generation of internal predictive models, which anticipate the sensory outcomes of motor activity and adjust for mismatches to ensure fluid execution (Wolpert et al., 1998). While this anticipatory correction mechanism is well established in motor domains, recent theories propose that similar computational strategies may be employed in non-motor contexts, enabling the cerebellum to support abstract reasoning, flexible behavior, and possibly emotional regulation. Once seen solely as a hub for movement coordination, the cerebellum is now considered integral to the broader neural architecture underpinning cognition and affective modulation (Stoodley and Schmahmann, 2009). Overall, the posterior lobe of the cerebellum seems crucial for cognitive functioning. Lesions selectively affecting lobules VI-X of the posterior but not the anterior lobe result in cognitive and affective deficits while leaving motor functioning mostly unimpaired (Stoodley and Schmahmann, 2009). Extensive research has furthermore delineated specific regions within the cerebellum that support distinct cognitive functions.

When following the traditional anatomical division of the cerebellum (Larsell, 1958), a plethora of studies have mapped cognitive functions to specific cerebellar regions.

The cerebellum has been shown to be involved in linguistic functions, which predominantly activate the right posterolateral hemisphere, especially lobules VI, Crus I, Crus II, and lobule VII (Stoodley, 2012; De Smet et al., 2013; Schmahmann, 2022). Tasks such as verb generation and verbal working memory have further shown activation in lobules VIIb-VIIIa besides lobule VI and Crus I (Stoodley, 2012).

Executive functions, including working memory, cognitive flexibility, and inhibitory control are consistently associated with lobule VI, Crus I, Crus II, lobule VIIb, lobule VIII, and lobule X (Stoodley, 2016; Clark et al., 2021; Schmahmann, 2022; Bègue et al., 2024). Working memory is associated with Crus I/II, lobule VI, and lobule VIIb, with right-lateralized activation for verbal working memory tasks (Stoodley, 2012; De Smet et al., 2013; Clark et al., 2021). Left Crus I/II have been identified as crucial regions for inhibitory control (Clark et al., 2021). Overall, activation during executive and attentional tasks was found to be bilateral in the posterolateral cerebellum (Buckner et al., 2011; Guell et al., 2018). Overlaps between those networks and cerebellar-vestibular regions point to these regions potentially encompassing spatial and orientational attention (Buckner et al., 2011; Baumann et al., 2015; Guell et al., 2018). Gray matter volume in Crus II and lobule X correspond to cognitive flexibility, processing speed, and working memory. Moreover, Crus I and lobule VI are specifically associated with working memory performance (Bègue et al., 2024). Visuospatial deficits and have been mapped to lesions in bilateral Crus I/II and right lobule VIII (Stoodley, 2016). Areas of the vestibulo-cerebellum correspond to not only balance and spatial orientation but further proposed to be involved in perceptual processing (Baumann et al., 2015). Tasks involving emotional and social cognition consistently show bilateral activation of the posterolateral cerebellar hemispheres, aligning with the general trend of cognitive processing being localized in the posterior cerebellum (Guell et al., 2018; Argyropoulos et al., 2020). The cerebellum’s involvement in memory functioning has been elaborated on in studies concerning episodic long-term memory during aging (Almeida et al., 2023). Crus I/II show activation during recall of correctly remembered words and posterior parts of lobule VI show a memory effect, specifically in semantic tasks (Fliessbach et al., 2007).

SCA34 presents with ataxic symptoms, impaired gait, limb incoordination, speech difficulties, and oculomotor abnormalities such as disrupted saccadic eye movements. Neuroimaging shows pontine and cerebellar atrophy with hypometabolism (Bourque et al., 2018). Despite these structural changes, the cognitive consequences of cerebellar and pontine degeneration in SCA34 remain underexplored. While individuals consistently show cerebellar atrophy on magnetic resonance imaging, similar to patterns seen across SCA subtypes (Nishide et al., 2024; Öz et al., 2024), the relationship between regional volume loss and cognitive deficits has not been systematically investigated. In addition to cerebellar involvement, SCA34 is also associated with pontine degeneration, detectable through advanced MRI techniques (Nishide et al., 2024). Emerging imaging modalities such as MR spectroscopy and quantitative susceptibility mapping have begun to uncover neurochemical alterations and abnormal iron deposition in SCA, offering new insights into disease mechanisms beyond structural atrophy (Öz et al., 2024). Research into SCA34 due to its rarity has limited detailed investigation. Existing reports suggest that the condition involves cognitive impairments, indicating cerebellar roles beyond motor control (Bourque et al., 2018; Beaudin et al., 2020). Such impairments are consistent with CCAS (Hoche et al., 2018). Beaudin (Öz et al., 2024) reported executive dysfunction and psychiatric symptoms in a family with SCA34. Yet, the complete neurocognitive profile and underlying mechanisms are still not well explored.

The aim of this study is to comprehensively characterize the neurological, neuroradiological, neurocognitive profiles, and psychiatric features of individuals with SCA34. In addition, we aim to explore neurocognitive functions in relationship to cerebellar morphology, specifically cortical thickness in specific cerebellar regions using high-resolution neuroimaging. Through analyses of morphometry, we seek to descriptively map structural changes to cognitive performance. Given the cerebellum’s involvement in SCA34, this work also aims to contribute to a broader understanding of the cerebellum’s role in cognition. We hypothesize that cognitive deficits and CCAS will coincide with observations of more severe cerebellar atrophy in the two patients.

## Materials and methods

### Participants

Four Swedish individuals diagnosed with SCA34 underwent comprehensive clinical assessments to characterize the neurological, neuroradiological, neurocognitive, and psychiatric manifestations associated with the disorder. Three of the four individuals successfully underwent high-resolution MRI, which allowed detailed analysis of cerebellar cortical thickness across multiple lobules. One individual was not eligible for MRI due to the presence of a pacemaker. In addition, one healthy control subject was included in the study to enable direct comparative analysis of cerebellar morphology with two of the affected individuals. The control subject was matched to the patients by sex, age (74 years), and years of education. A demographically matched control subject was not available for P4. Detailed descriptions of the neurocognitive evaluation protocol and test results are available in Supplementary materials S1, S2.

### Standard protocol approvals, registrations, and patient consents

This study was approved by the Swedish Ethical Review Authority (Dnr 2022-01799-01). Participants showing signs of mental disorder, suicidal thoughts, or concerning test results were referred to appropriate care. All the human data in the study was collected in line with the 1964 Helsinki Declaration. Furthermore, the data collected was handled in accordance with the Swedish Patient Data Act (2008:355) as well as the General Data Protection Regulation (GDPR). All participants were informed that the results would be published but that they would remain anonymous. The manuscript contains no identifiable information.

### Molecular genetic analysis

The SCA34 diagnosis was verified by Next-Generation Sequencing (NGS) prior to the study which was performed on EDTA blood collected from the ataxia individuals to verify the SCA-diagnosis.

### Cut-off criteria and normative data for neurocognitive and motor tests

To define cognitive and motor deficits, we applied cut-offs based on normative data. Scores 1 standard deviation (SD) below the premorbid level were considered indicative of a mild deficit, while scores 1.5–2 SD below were classified as significant impairments. These thresholds were applied across all administered tests to categorize participants’ performance as normal, mildly reduced (deficit), or significantly reduced (impairment) relative to age- and education-matched norms, following recommendations from Jak et al. (2009) and Tanner-Eggen et al. (2015) and in line with common neuropsychological practice (Goldman et al., 2013; Munthe-Kaas et al., 2020). Normative data for the Emotion Recognition Task of Cambridge Neuropsychological Test Automated Battery (CANTAB), Finger Tapping, and Prism Adaptation tasks are not available in the Swedish population. Therefore, we established primary normative data using our own sample of 96 healthy individual which served as the reference for defining performance thresholds. The control sample ranged in age from 18 to 85 years (38.78 ± 15.78) and included 54.5% female participants, providing a broad representation of adult age groups. Educational attainment was recorded for all participants and categorized according to completed years (15.42 ± 2.36). Raw test scores were converted to z-scores based on the mean and standard deviation of the control sample. Z-scores were then used for standardization, allowing performance to be interpreted relative to the control group while accounting for the distribution of scores across the sample.

### Verbal functions

#### Vocabulary, subtest of Wechsler adult intelligence scale (WAIS-IV)

This subtest assesses basic semantic knowledge, verbal concept formation, verbal expression and overall language development. Successful performance requires both auditory comprehension and verbal expressive abilities (Wechsler, 2008).

#### Letter and category fluency, subtests of verbal fluency test of Delis-Kaplan executive function system

These subtests measure functions related to verbal abilities and executive processes, as well the ability to generate words fluently in an effortful, phonemic format (Letter Fluency) and from overlearned concepts (Category Fluency). We selected for analysis Letter and Category Fluency which minimizes the influence of executive functions compared to Category Switching (Delis et al., 2001).

#### Boston naming test

This is a brief language assessment in which the examinee names line drawings of objects that become progressively less common in everyday language. The test is primarily designed to assess confrontation naming (Goodglass et al., 1983).

### Executive functions

#### Number-letter switching, trail making test of Delis-Kaplan executive function system

This subtest primarily measures cognitive flexibility, it also evaluates inhibitory control, visuomotor speed, working memory, and task-switching efficiency (Delis et al., 2001).

#### Inhibition, color words interference test of Delis-Kaplan executive function system

The test has four conditions, of which the third was of interest in this study. This condition measures inhibition (Delis et al., 2001) and requires the inhibition of the learned behavior of reading words aloud as they are written. The variable selected for the analysis was total time in seconds.

#### Digit span, subtest of Wechsler adult intelligence scale (WAIS-IV)

The test was administered to assess auditory attention, working memory, and short-term memory capacity (Wechsler, 2008). The subtest includes three conditions: *Digit Span Forward*, *Digit Span Backward*, and *Digit Span Sequencing*. In the *Forward* condition, participants are required to repeat sequences of numbers in the same order as presented; in the *Backward* condition, they repeat the sequences in reverse order; and in the *Sequencing* condition, they recall the numbers in ascending numerical order. The primary variable selected for analysis was the total raw score across all three conditions, which provides an overall measure of auditory-verbal working memory efficiency.

#### Spatial span, Cambridge neuropsychological test automated battery

All CANTAB tasks were administered on an iPad (9th generation; 10.5-inch screen). *Spatial Span* (SSP) is a computerized version of the Corsi Block Tapping Task (Milner, 1971) and measures visuospatial working memory. The variable selected for the analysis was *SSPFSL* from Standard Forward 2.0 (the longest sequence of boxes successfully recalled by the subject, applicable to Forward variants only) (CANTAB® [Cognitive Assessment Software], 2019).

### Attention and processing speed

#### Symbol search and coding, subtest of Wechsler adult intelligence scale (WAIS-IV)

Both subtests measure sensorimotor integration, visual scanning, and processing speed (Wechsler, 2008; Lichtenberger and Kaufman, 2012). The *Coding* subtest primarily measures incidental learning and visual-motor coordination whereas the *Symbol Search* subtest primarily measures visual discrimination and visual scanning.

#### Visual scanning, trail making test of Delis-Kaplan executive function system

This subtest measures visual scanning ability, visual and selective attention. This specific condition isolates visual scanning ability by removing the need for motor sequencing or number-letter alternation (Delis et al., 2001).

### Learning and memory

#### Rey auditory verbal learning test

It primarily measures verbal learning and memory, as well immediate memory span, learning across trials, delayed recall and recognition memory (Rey, 1964; Vakil and Blachstein, 1997). The Rey Auditory Verbal Learning Test (RAVLT) involves repeating a 15-word list over five trials. A second list is briefly introduced, then participants recall the original list again, both immediately and after 20–30 min. Scoring is based on correct recalls and memory retention across trials.

#### Paired associated learning and pattern recognition memory, Cambridge neuropsychological test automated battery

They assess visual episodic memory and visual recognition memory (CANTAB® [Cognitive Assessment Software], 2019). The variables selected for the analysis were *PALTEA28* from Standard Extended (Total Errors: the number of times the subject chose the incorrect box for a stimulus on assessment problems), *PALFAMS28* (First Attempt Memory Score: the number of times a subject chose the correct box on their first attempt when recalling the pattern locations), *PRMPCI* from Standard 18 Extended (the number of correct patterns selected by the subject in the immediate forced-choice condition) and *PRMPCD* (Percent Correct Delayed: the number of correct patterns selected by the subject in the delayed forced-choice condition).

### Visuospatial perception and visuospatial abilities

#### Block design, subtest of Wechsler adult intelligence scale (WAIS-IV)

This test assesses visuoconstructive functions, visuospatial perception, perceptual reasoning, and organization. It also requires visuomotor coordination. We selected for analysis *Block Design without time bonus* which minimizes the influence of speed. It is better for isolating spatial reasoning and constructive skills, especially in individuals with motor, neurological or speed-related impairments (Wechsler, 2008).

#### Copy task, visual reproduction II of Wechsler memory scale III

This test measures primarily visuospatial abilities, visuospatial construction, the ability to perceive spatial relationships and accurately reproduce visual information. Fine motor skills are also involved in accurate drawing. The scoring criteria measure accuracy and spatial positioning (Wechsler, 1997).

#### Cube analysis and silhouettes, subtests of visual object and space perception battery

Cube Analysis measures visuospatial perception and visual discrimination (Warrington and James, 1991). Silhouettes measure visuospatial perception and the ability to recognize common objects and animals depicted from unconventional perspectives.

### Social cognition

#### Emotion recognition task, Cambridge neuropsychological test automated battery

Emotion Recognition Task (ERT) assesses a general deficit in emotion recognition, particularly where global emotion detection is of interest, usually in groups where social interaction is impaired (Kessels et al., 2014). The variable selected for the analysis was *ERTTH* (the total number of correctly identified emotions by the participant across all trials). The ERT task used for this study included 48 trials (short version) (CANTAB® [Cognitive Assessment Software], 2019).

### Cerebellar and motor functions

#### Finger tapping

This test measures sensorimotor synchronization and the ability to maintain rhythm and can be used to assess motor control and the integrity of the neuromuscular system (Gustafsson et al., 2023). We evaluated isochronous serial interval production (Madison, 2006), that has been linked to cerebellar function (Rivkin et al., 2003). The variables selected for the analysis were production mean (ms), local (ms), and drift (ms).

#### Prism adaptation

This test measures sensorimotor coordination following changes in visual input. Previous studies demonstrate a relationship between performance in prism adaptation and cerebellar function (Pisella et al., 2005; Küper et al., 2014). For the analysis we examined the error in centimeters on the first trial after putting the glasses on (trial 11), the error on the first trial after taking the glasses off (trial 21) and the average absolute error across all 30 trials.

#### Motor speed, trail making test of Delis-Kaplan executive function system

This subtest measures motor speed without cognitive load. It provides a direct, isolated measure of motor demands (Delis et al., 2001). The task involves precise line-tracing, which taps into fine motor coordination and manual dexterity.

### Clinical scales

#### Hospital anxiety and depression scale

The Hospital Anxiety and Depression Scale (HADS) is a self-report questionnaire that contains 14 items, 7 items measuring cognitive and emotional aspects of depression, specifically anhedonia, and the remaining 7 items focusing on cognitive and emotional aspects of anxiety (Zigmond and Snaith, 1983). High scores indicate greater severity. Both HADS-A (α = 0.87) and HADS-D (α = 0.81) have high reliability.

#### Insomnia severity index

The Insomnia Severity Index (ISI) is a 7-item self-report questionnaire assessing insomnia severity and impact in the last 2 weeks (Bastien et al., 2001). High scores indicate greater severity. ISI has high reliability (α = 0.79).

#### Scale for the assessment and rating of ataxia

The *SARA* is a clinical scale measuring the severity and progression of cerebellar ataxia (Schmitz-Hübsch et al., 2006; Kim et al., 2011). It is a standardized, quantitative scale that assesses motor function through a series of performance-based tasks. The scale includes eight items such as gait, stance, sitting, speech disturbance, and limb coordination (e.g., finger chase, nose-finger test, fast alternating hand movements and heel-shin slide), with each task scored on a defined scale.

### Computed tomography

Non-contrast computed tomography (CT) scans were obtained to provide supplementary structural information, particularly in cases where magnetic resonance imaging (MRI) was unavailable or contraindicated like for (P3) (Diedrichsen et al., 2009; Kaipainen et al., 2021). CT images were systematically reviewed by a Senior Neuroradiologist for global and regional brain atrophy, white matter alterations, and other structural abnormalities relevant to the study objectives. CT-based visual ratings of cortical and medial temporal atrophy have been shown to correlate strongly with MRI-derived measures (Kaipainen et al., 2021; Sánchez-Moreno et al., 2025), supporting the utility of CT as a practical alternative for evaluating brain atrophy in clinical and research settings.

### Magnetic resonance imaging

The images were acquired using the 7 T MRI scanner (Philips, Best the Netherlands) of the Swedish National 7 T MR facility, Lund University and Skåne University Hospital. The high field strength provides the high spatial resolution required for an accurate and reliable quantification of cerebellar cortical thickness, which is not sufficiently attainable at 3 T. An 8-channel transmit, 32-channel receive head coil was used (Nova Medical, Wilmington MA, United States). The consensus recommendations on MR screening of ataxic individuals by the Ataxia Global Initiative Working Group on MRI Biomarkers were followed (2024). For highly detailed, T1-weighted imaging of the cerebellum, we employed a 3D MP2RAGE sequence with 0.5 mm isotropic resolution (Marques et al., 2010). This data provided a basis for subsequent tissue segmentation and cortical thickness estimation. A full description of the MRI protocol is presented in Supplementary material S3.

### Magnetic resonance analysis

The MRI analysis was intended primarily as a methodological test rather than a definitive group comparison. The cerebellar-focused MP2RAGEs were processed using the Nighres toolbox (Huntenburg et al., 2018), which is optimized for ultra-high-resolution 7 T images. The images were denoised and segmented following Priovoulos and Bazin’s (2023) pipeline.^1^ After denoising and reconstruction of bias-free T1-weighted images (UNI) and quantitative T1-maps (T1), which were co-registered to MNI space. Using a segmentation pipeline (Huntenburg et al., 2018; Priovoulos and Bazin, 2023), tissue in the cerebellum was classified as gray matter (GM), white matter (WM) or cerebrospinal fluid (CSF). This tissue segmentation allows for building a 3D model of the cerebellar cortex, and subsequent analysis of cortical shape and thickness. The lobules of interest were analyzed as ROIs. Regional cortical thickness was calculated by averaging the thickness values within each functional ROI, allowing assessment of thickness variation across functional parcellations of the cerebellum. Cortical thickness was selected due to its association with granule layer density, which is decreased in individuals suffering from ataxia (Pascual-Castroviejo et al., 1994). The cerebellar cortical thickness values were compared between the two scanned individuals and control. A complete description of the analysis process is given in Supplementary material S3.

## Results

### Neurological evaluation

For detailed information on neurological findings refer to Table 1 and to Supplementary material S2. All four related individuals carry the same genetic variant in the *ELOVL4* gene: c.511A > C, p.(Ile171Leu). Neurologically, all individuals exhibited gaze-evoked nystagmus (GEN) and ataxia affecting gait as well as both upper and lower limbs. Mild dysarthria was present in all individuals. All except P4 showed hypometric saccades, low-gain and saccadic smooth pursuit. P4 had restricted upgaze. P2 also presented with increased vestibulo-ocular reflex (VOR) gain, strabismus, unilateral ptosis, and diplopia. Tendon reflexes were decreased only in P1 and in P2. Notably, extrapyramidal signs were absent in all four individuals.

**Table 1:** Detailed neurological and psychiatric findings in SCA 34 individuals.

ID	P1	P2	P3	P4
Age of last evaluation	70	74	74	50
Reported age of onset	50	41	61	46
Ataxia upper and lower limbs	++	+++	+++	+++
Dysarthria	+	+	+	+
SARA score (2024)	12.5	17.25	21.5	22
Nystagmus	+	+	+	+
Hypometric saccades and saccadic pursuit	+	+	+	−
Tremor	−	−	−	−
Strength	N	N	N	N
Sensoric function	N	N	N	N
Evolution	Slow	Slow*	Slow*	Slow*
Skin lesions	+	+	+	+
Others	dDTR, OSAS, SZ	CVD, CVH, dDTR, MCI, OSAS	CVD, SZ	MCI, SCZ
Self-report clinical scales (2025)				
HADD (depression)	0	4	5	4
HADA (anxiety)	5	2	8	11
ISI (sleep)	2	0	17	5

− = absent; + = present/mild; ++ = moderate; +++ = severe; * = uses a walker; N = normal; NA = not available. Clinical and diagnostic terms: CVD = cardiovascular disease; CVH = complex visual hallucinations; dDTR = decreased deep tendon reflex; MCI = mild cognitive impairment; OSAS = obstructive sleep apnea syndrome; SCZ = schizophrenia; SZ = epileptic seizures. HADS = Hospital Anxiety and Depression Scale: Depression index (0–6 = not depressed, 7–10 = low mood, >10 = risk of depressive disorder) and Anxiety index (0–6 = no clinically significant anxiety, 7–10 = mild to moderate anxiety, >10 = possible anxiety disorder). ISI = Insomnia Severity Index: 0–7 = no clinically significant sleep difficulties, 8–14 = subthreshold insomnia (cut-off <10; 11–14 = mild insomnia), 15–21 = clinical insomnia (moderate), 22–28 = clinical insomnia (severe).

### Magnetic resonance imaging

Three out of four individuals (P1-P2-P4) with SCA34, along with one matched control, underwent 7 T MRI scanning. One individual with SCA34 (P3) could not be scanned due to a pacemaker implantation and instead underwent computed tomography. For the MR analysis, we included two individuals with SCA34 (P1 and P2) and one matched control. We excluded from MR analysis P4 because we could identify any matched control. No additional control subjects matched for sex, age, and education could be identified for the MRI analysis. Detailed description of the results can be found in Table 2 and Supplementary material S2.

**Table 2:** Detailed neuroradiological findings in SCA 34 and healthy control.

Neuroradiological parameter	P1	P2	P3	P4	Control
Magnetic resonance date	2025/June	2025/June	No	2025/June	2025/June
Computed tomography date†	NA	NA	2025/January	NA	NA
Atrophy grade					
Global cortical atrophy	1	**1–2**	1†	1	**1–2**
Parietal right	1	1	1†	**2**	1
Parietal left	1	1	1†	**2**	1
Frontal atrophy	1	1	1†	1	1
Medial temporal right	1	1	1†	1	1
Medial temporal left	**2**	1	1†	1	1
Cerebellum right	**2**	**2**	**2**†	**3**	1
Cerebellum left	**2**	**2**	**2**†	**3**	1
Vermis	**2**	**2**	**3**†	**3**	0
Middle cerebellar peduncle right	**2**	1	**2**†	**2**	0
Middle cerebellar peduncle left	**2**	1	**2**†	**2**	0
Superior cerebellar peduncle right	1	**2**	**2**†	**3**	0
Superior cerebellar peduncle left	**2**	**2**	**2**†	**3**	0
Pons	1	1	1†	1	0
Mesencephalon	**2**	1	1†	0	0
White matter hyperintensities (Fazekas)	1	1	**3**†	1	**3**
Lacunar infarcts	0	0	0†	0	0
Comparison with previous imaging	0	0	0†	0	0

† = data derived from computed tomography scan; included for descriptive purposes only and not directly compared with magnetic resonance imaging metrics; NA = not available; No = the individual could not undergo 7T MRI scanning due to the presence of a pacemaker; Atrophy grading classifies as follows: Grade 1 = mild, Grade 2 = moderate, and Grade 3 = severe; Numbers marked in bold indicate greater atrophy; P1 = Individual 1 with SCA34; P2 = Individual 2 with SCA34; P3 = Individual 3 with SCA34; P4 = Individual 4 with SCA34.

**Figure 1:**
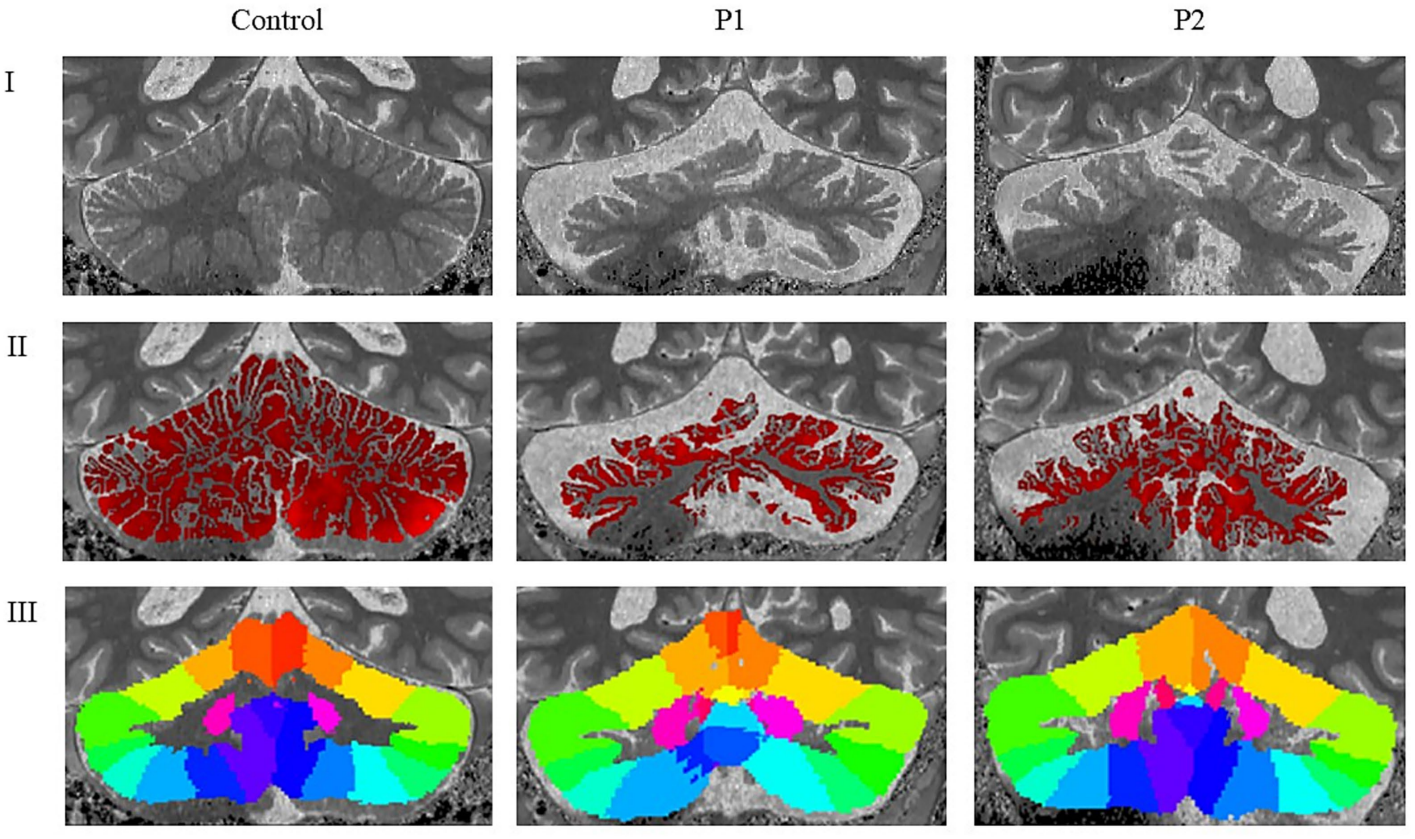
Neuroradiological findings in healthy control, P1 and P2. I: Cropped and rescaled T1-weighted anatomical image; II: Cerebellar cortical thickness map derived from the T1-weighted image; III: Cerebellar lobular atlas warped to the subject’s native anatomical space; P1 = Individual 1 with SCA34; P2 = Individual 2 with SCA34.

Results were broadly consistent across P1 and P2, revealing mild small vessel disease (Fazekas grade 1), mild to moderate global cortical atrophy, and mild medial temporal and parietal atrophy. Most prominent, however, was the presence of moderate bilateral cerebellar atrophy—including involvement of the vermis—and atrophy of the middle and superior cerebellar peduncles. Mild pontine atrophy was also observed in P1 and P2. No lacunar infarctions, large infarcts, or other focal lesions were detected. Overall, the imaging findings reflect moderate generalized neurodegeneration, with a predominant cerebellar pattern consistent with the clinical manifestations of SCA34. Results of the two analyzed individuals (P1-P2) are outlined in relation to cerebellar morphology and further connected to the body of research presented earlier. Results are presented domain-wise, assessing cortical thickness in cerebellar regions, as defined through lobular anatomical parcellation. Despite the cerebellar-targeted phase setting, the images exhibited signal loss at the base of the cerebellum. Due to this, the segmentation was imperfect on the right side (see Figure 1); therefore, results from the inferior right cerebellum are excluded from analysis. Affected are the following lobules: right Crus II, right lobule VIIb, right lobule VIIIa, right lobule VIIIb and right lobule IX. Compared to the control, both individuals showed reduced cerebellar cortical thickness across nearly all anatomical regions (see Table 3). An exception was observed in P1, where thickness in the right Crus I exceeded the control value.

**Table 3:** Cerebellar cortical thickness for cerebellar anatomical regions.

Cerebellar region	Control	P1	P2
Left lobule VI	2.653	1.902	1.348
Right lobule VI	2.578	1.905	1.145
Left Crus I	2.269	1.754	1.088
Right Crus I	1.814	2.014	1.102
Left Crus II	3.898	1.648	1.635
Left lobule VIIb	5.743	2.318	2.171
Left lobule VIIIa	5.063	1.832	2.531
Left lobule VIIIb	5.795	1.955	2.379
Left lobule X	5.947	1.711	0.83
Right lobule X	5.115	0.994	0.967

This table presents the distribution of cerebellar cortical thickness (in millimeters) across various cerebellar anatomical regions.

P3 exhibited severe deep white matter changes (Fazekas grade 3), while P4 showed mild deep white matter changes (Fazekas grade 1), both P3 and P4 exhibited mild global cortical atrophy (GCA 1), and mild medial temporal atrophy (MTA 1). P3 showed mild parietal atrophy (PA 1), while P4 had moderate parietal atrophy (PA 2). In both individuals, cerebellar atrophy was evident, with P3 displaying moderate bilateral cerebellar atrophy, including the vermis, and P4 showing severe bilateral cerebellar atrophy with more pronounced vermian involvement. Both cases demonstrated moderate atrophy of the middle cerebellar peduncles, with P4 also presenting severe atrophy of the superior cerebellar peduncles, compared to moderate atrophy in P3. Mild pontine atrophy was noted in both patients. No lacunar infarctions, major infarcts, or other focal lesions were identified. Overall, the findings indicate mild generalized cerebral atrophy and extensive cerebellar degeneration in both individuals, including involvement of the cerebellar peduncles and vermis. Small vessel disease (SVD) was mild but widespread in P3 and mild in P4.

### Linking cerebellar morphometry and neurocognition

#### Verbal functions

Verbal functions are differentially afflicted across P1 and P2. In P1 verbal functions are partly decreased. Results show decreased performance in semantic knowledge, verbal comprehension and verbal expression. Furthermore, confrontational word retrieval, phonemic and semantic fluency are decreased. In contrast, P2 presents more severe decreases in verbal functions. Results show impairments for verbal comprehension, semantic knowledge and verbal expression as well as decreased performance in confrontational word retrieval. Phonemic and semantic fluency are heavily impaired also due to executive dysfunctions.

Cerebellar regions associated with language processing exhibit morphometric differences between the two individuals. Right lateralized language-associated structures such as the right lobule VI, right Crus I and right Crus II seem to be more reduced in P2 compared to P1 (see Table 3). These regions are known to support phonological processing, verbal fluency, and syntactic comprehension (Stoodley, 2012; Schmahmann, 2022).

#### Executive functions

P1 and P2 differ in performance on the executive functions. P1 exhibited relatively preserved cognitive flexibility in some tasks while highly impaired in others (see Supplementary material S2), and decreased performance in inhibitory control, auditory and visuospatial working memory tasks. P2, in contrast, demonstrated significant impairments across all three functions, with severe impaired performance. Furthermore, both individuals showed a high number of set loss errors in the verbal fluency task reflecting reduced flexible retrieval from semantic networks, pointing to deficits in inhibition and short-term memory.

Inhibition has been found to be lateralized in the left Crus I and left Crus II, both of which are thinner in P2, with a bigger difference for the left Crus I. Cognitive flexibility, processing speed and working memory have been found as a cluster in Crus II and lobule X, here again, P1 shows higher thickness values in all areas. Additionally, small differences were observed in right lobule X and left Crus II. Working memory tasks are associated with Crus I, Crus II, lobules VI and VIIb, which all are decreased in P2 as compared to P1. Consistent with language-associated lateralization, right lobule VI and right Crus I have been shown to be implicated in verbal working memory tasks and were decreased in P2 relative to P1, in line with their neurocognitive performance (see Supplementary material s2). Moreover, lobule VIII is linked to executive functions and showed thinning in P2 as compared to P1. This is not reflected in the overall pattern observed in the two individuals.

#### Attention and processing speed

Attention and processing speed were decreased in P1, with decreased performance in visual scanning and visual attention, as well as slight decreases in complex attention and processing speed (see Supplementary material S2). P2 showed severe impairments on all attentional functions as well as processing speed. Processing speed has been associated with Crus II and lobule X, which, as stated above, were both decreased in P2 compared to P1 (see Table 3).

#### Learning and episodic memory

In P1 visual and verbal memory are decreased. The results show decreased performance for learning, immediate recall, and recognition. Low performance in delayed recall was only observed in visual while it was preserved in verbal episodic memory tasks. In contrast, P2 exhibits impairments across both visual and verbal episodic memory tasks. Learning abilities are slightly decreased in P1 and severely impaired performance in P2. The results show impairments in learning, immediate recall, delayed recall and recognition (see Supplementary material S2). Memory recall has been associated with Crus I and Crus II, here P1, exhibited higher cortical thickness compared to P2. Moreover, right lobule VI was thinner in P2 than in P1 (see Table 3).

#### Visuospatial perception and visuospatial abilities

Performance on tests assessing visuospatial abilities and visuospatial perception again revealed differences between the two individuals. P1 functions were decreased. The individual demonstrated slight decreases in performance in visual spatial processing, problem solving and visual motor construction as well as object and animal recognition (see Supplementary material S2).

Impairments were observed in the figure-copy tasks. However, these impairments might partly result from motor impairments as assessed in the Motor Speed task. Relative to P2, P1’s visuospatial functions are more intact. P2’s performance on visuospatial abilities and visuospatial perception is decreased as well. The individual showed impairments for visuo-constructive problem solving, deficits in visuospatial perception, visual discrimination, and object and animal recognition from unconventional perspectives. Severe impairments were observed in the figure-copy task, which could also stem from observed motor impairments.

Cerebellar structures associated with spatial cognition include lobule VI and bilateral Crus I/II, (Stoodley, 2016). Taking a closer look at lobule VI, which has been pointed out to be highly implicated in visuospatial abilities, reveals lower cerebellar cortical thickness in P2 as compared to P1. Crus I is bilaterally lower and left Crus II, due to data dropout, unilaterally lower in P2 relative to P1. Perceptual tasks have been associated with regions in the previously stated areas of the vestibulo cerebellum and decreases in perceptual performances provide mixed results in these brain regions with (see Table 3).

### Neurocognitive and motor performance findings

Given that our neurocognitive findings are based on a small sample of only four individuals with SCA34, it is difficult to draw strong conclusions regarding the homogeneity of the cognitive profile associated with this condition. The presence of comorbidities, along with individual differences in age, sex, and educational background, introduces additional variability that may influence neurocognitive performance. These factors can significantly modulate cognitive outcomes and limit the generalizability of the results. Despite the small sample size, our results reveal several shared neurocognitive deficits across all four individuals. Specifically, each participant demonstrated impairments in verbal phonemic fluency, indicating reduced lexical access and executive control over word retrieval. There was also a consistent reduction in inhibitory control and processing speed, as well as deficits in visual search and visual attention. In the cognitive domain of memory, all individuals showed difficulties in verbal and visual episodic memory acquisition and recognition, suggesting disruptions in both encoding and retrieval processes, neuroradiological findings also show atrophy in medial temporal lobes in all individuals. Additionally, visuospatial and visuoconstructive abilities were robustly impaired, along with deficits in visual perception—particularly in the recognition of animals and objects, pointing toward higher-order visual processing difficulties.

For the Emotion Recognition Task, normative data in the Swedish language are not yet available. Therefore, normative data were derived from our sample of 101 healthy controls. Despite this, all individuals in the study demonstrated deficits in this function, which contribute to adequate social cognition functioning (Meyer and Kurtz, 2009; Razmjooei et al., 2024).

Sensorimotor functions were also compromised, as indicated by significantly reduced motor speed measured using the Condition 5 of Trail Making Test (D-KEFS) (Delis et al., 2001). Gross motor coordination and visuospatial adaptation, assessed with the Prism Adaptation task, were severely impaired. In contrast, sensorimotor synchronization was less affected in the patients, possibly due to partially preserved fine motor abilities.

Taken together, these findings suggest a widespread pattern of cognitive and sensorimotor involvement in SCA34 that affects multiple domains beyond the traditionally expected cerebellar motor symptoms. As such, while our observations offer valuable preliminary insights into the cognitive features of SCA34, larger and more controlled studies are necessary to determine whether a consistent neurocognitive pattern can be reliably identified in this rare patient population.

## Discussion

Our findings revealed neurocognitive impairments consistent with CCAS, a condition characterized by deficits in executive functioning, verbal processing, visuospatial skills, and affect regulation (Schmahmann and Sherman, 1998). In addition to the core features of CCAS, we identified pronounced impairments in both visual and verbal episodic memory, as well as in emotion recognition, a critical component of social cognition. These additional deficits suggest a broader pattern of cerebellar involvement in cognitive and affective processes than traditionally appreciated.

Impairments in episodic memory, particularly in both visual and verbal domains, may reflect disruptions in cerebello-cortical circuits that extend beyond the classical fronto-cerebellar loops typically associated with executive dysfunction. However, it should also be noted that atrophy was observed in the medial temporal lobe, a region critically involved in episodic memory processing. Emerging evidence suggests that the cerebellum contributes to memory processes through its connections with the hippocampus and parietal regions (Andreasen et al., 1999; Almeida et al., 2023), which may help explain the observed memory deficits in our individuals. This aligns with recent studies indicating that cerebellar damage can affect memory consolidation and retrieval, even in the absence of overt hippocampal pathology. Moreover, we observed deficits in emotion recognition task. Social cognition, the ability to perceive, interpret, and respond to social cues, relies heavily on accurate emotion recognition. Deficits in this domain have been increasingly documented in patients with cerebellar damage, further supporting the cerebellum’s role in modulating affective and interpersonal functioning (Adamaszek et al., 2014; Van Overwalle et al., 2020; Pierce et al., 2023). These findings are consistent with the hypothesis that the cerebellum serves as a modulatory hub not only for motor control but also for higher-order cognitive and affective processes. The presence of both CCAS-related symptoms and additional impairments in episodic memory and emotion recognition underscores the importance of comprehensive neurocognitive assessment in individuals with cerebellar pathology.

While depressive symptoms were not observed in our cohort, individuals did exhibit mild symptoms of anxiety and sleep disturbances. Other psychiatric conditions were not evident during clinical evaluations. These non-motor features, though often overshadowed by the more apparent motor impairments in cerebellar ataxias, are increasingly recognized as important contributors to patient well-being. Anxiety and disrupted sleep patterns can exacerbate cognitive difficulties, reduce coping capacity, and negatively affect daily functioning, even when mood symptoms such as depression are absent.

The presence of these symptoms highlights the need for comprehensive and routine clinical monitoring in individuals with cerebellar pathology. Subtle, heterogeneous cognitive and psychiatric symptoms in cerebellar ataxias often remain unrecognized without targeted evaluation. The analyzed MRI results reveal widespread cortical thinning in P1 and P2 relative to the control, which confirm atrophy in both individuals. P2 consistently exhibited greater reductions and broader cognitive deficits than P1, indicating a correspondence between the severity of structural and functional changes. The pattern of cognitive impairments aligns with reductions in cortical thickness within cerebellar regions known to subserve those functions. Cognitive functions, including language, executive functions, attention, and visuospatial processing, demonstrated alignment with regional cerebellar thinning in areas known to support these functions. An exception to this alignment was found in P1’s preserved performance only in delayed recall in verbal episodic memory. Observed atrophy in Crus I, left Crus II, and lobule VI did not correspond with this preserved performance. Moreover, P1 higher thinning in lobule VIII slightly aligns with the performance in executive functions. Both findings could potentially be explained by compensatory mechanisms or methodological limitations. Additionally, it is important to note that extracerebellar atrophy was also observed, as well observed particularly in medial temporal lobe regions, which are especially relevant to the preserved performance in verbal delayed recall. This underlines the complexity of cerebellar structure–function mapping and the need for larger samples to disentangle consistent correspondences from individual variability.

Thinning in the right-lateralized lobule VI and Crus I aligned with language performance. This aligns with previous reports of right-lateralized contributions of the posterolateral cerebellum to phonological processing, fluency, and syntactic comprehension (Stoodley and Schmahmann, 2009; Schmahmann, 2022). Executive dysfunction impairments were most pronounced in P2, aligning with greater thinning in Crus I/II, and lobule X, consistent with prior findings linking these regions to inhibition, flexibility, and working memory. Attention and processing speed were reduced in both individuals, aligning with decreased cortical thickness in Crus II and lobule X. Visuospatial impairments corresponded to observed thinning in lobule VI, Crus I and II.

In both individuals, CCAS-consistent structure–function mapping linked executive deficits to Crus I/II and lobules VI and X, language to right Crus I and lobule VI, and visuospatial deficits to lobule VI, Crus I, and Crus II. Memory deficits in verbal and visual episodic encoding further support cerebellar involvement in executive and episodic memory functions. Beyond its alignment with the CCAS, this finding emphasizes the cerebellum’s domain-general computational role (Schmahmann, 1991). The results of the two scanned individuals fit the prevalence of Crus I, Crus II and lobule VI across different functions beyond domain-specific associations.

SARA scores indicate moderate ataxia in P1 and severe ataxia in others align with behavioral and neurocognitive performance. Specifically, impairments observed in *Prism Adaptation* and *Finger Tapping* point to disruptions in cerebellar sensorimotor processing, visuospatial adaptation and synchronization. Additionally, performance on the *Motor Speed* condition of the Trail Making Test from D-KEFS indicates impairments in pure motor speed, independent from cognitive load. This measure isolates motor speed from other cognitive demands, this neurocognitive task holds potential for further use in cerebellar research, particularly in distinguishing motor impairments from higher-order cognitive dysfunction. Its application in this context may represent a novel approach to assessing cerebellar-related motor slowing in several neurodegenerative disorders.

## Limitations

We acknowledge the small sample size as a key limitation of this study. However, SCA34 is a rare form of spinocerebellar ataxia (Alshimemeri et al., 2024; Nishide et al., 2024), with only a limited number of genetically confirmed cases reported worldwide. To our knowledge there are no other cases reported in Sweden. As in many rare disease studies, the small cohort, limited to two families, not capture the full SCA34 phenotype. Comorbidities further complicate interpretation, as some findings may reflect individual variation rather than core disease features.

Despite these limitations, the observed neurocognitive impairments are likely not specific to a particular mutation but instead reflect features of cerebellar motor syndrome. These cognitive deficits appear to stem from cerebellar degeneration, aligning with established links between cerebellar structure and cognitive function. This work was designed as a preliminary or pilot study, and the MRI analysis was intended primarily as a methodological test rather than a definitive group comparison. However, due to the case study design and limited generalizability, these conclusions should be interpreted with caution. Additionally, only three participants were able to perform MRI and we have detailed analysis only of two of them. Future studies should include a larger number of control subjects to strengthen and validate our findings.

Technical limitations also affected data quality and analysis. Structural MRI of the cerebellum presents unique challenges due to its anatomical complexity and frequent exclusion from standard cognitive imaging protocols. In this study, the cerebellar imaging pipeline was still under development at the time of data acquisition. As a result, data loss occurred in inferior regions of the cerebellum, particularly in individuals with larger head sizes, due to scanner constraints and segmentation difficulties. These limitations can be addressed in future studies using refined parallel transmission and local B0-shimming methods currently being developed.

High levels of cerebellar atrophy and increased head motion further impeded clean segmentation, introducing alignment errors during atlas-based registration. These registration tools, often developed on healthy populations, are less robust in neurodegenerative contexts and required partial manual correction. Consequently, usable data from the inferior right cerebellum were limited, reducing the explanatory power of the findings in that region. Additionally, the inferential nature of MRI-based structure–function mapping must be acknowledged. Given the inter-individual variability in functional topography, associations between regional atrophy and cognitive deficits are probabilistic rather than deterministic. Structural MRI alone cannot definitively establish causality between atrophy and cognitive impairment, as both may result from or be mediated by broader disruptions in cerebello-cortical networks. Finally, while ultra-high field 7 T MRI offers significant advantages in spatial resolution and contrast, it is also more susceptible to B0 and B1 field in homogeneities and imaging artifacts, which may have further impacted image quality in this study.

## Conclusion

This study provides a comprehensive neurological, neuroradiological, neurocognitive characterization of SCA34, confirming the pathogenic role of *ELOVL4* variants. High-resolution 7 T MRI revealed structural cerebellar alterations corresponding with distinct neurocognitive impairments, including deficits consistent with CCAS, as well as novel impairments in learning and episodic memory, social cognition (e.g., emotion recognition), and psychiatric symptoms such as anxiety and sleep disturbances. The findings highlight the cerebellum’s crucial role in higher-order functions, linking lobular cortical thinning to cognitive impairments in SCA34 and supporting the need for cognitive and psychiatric assessments in clinical care.

## Future research

Further research is needed to elucidate the specific role of *ELOVL4* in cerebellar function, in order to clarify the underlying pathological mechanisms contributing to the selective cerebellar degeneration observed in SCA34. Given the rarity of SCA34, larger and more diverse individual cohorts are essential to determine whether the neurocognitive and neuroimaging patterns identified in this study can be replicated. Multi-center collaborations are necessary to achieve sufficient sample sizes and improve the generalizability of findings. Longitudinal studies tracking both cerebellar and cortical changes in relation to cognitive performance over time are particularly valuable for delineating the temporal dynamics of disease progression. Integrating structural imaging with functional MRI, both task-based and resting-state fMRI as well as diffusion MRI, could provide insight into network-level disruptions and offer a more direct understanding of how cerebellar dysfunction contributes to cognitive and psychiatric symptoms. Such approaches may also help uncover compensatory mechanisms and functional plasticity within cerebellar networks. The imaging and segmentation pipeline used in this study could serve as a valuable foundation for future research in larger SCA cohorts. With expanded datasets, deep learning–based segmentation methods could supplement or replace traditional atlas-based approaches, improving accuracy in cases with pronounced atrophy and enabling more robust lobular parcellation. Collectively, these directions will help advance our understanding of cerebellar contributions to cognition and emotion, while refining diagnostic and monitoring tools for cerebellar ataxias.

## Data Availability

The original contributions presented in the study are included in the article/Supplementary material, further inquiries can be directed to the corresponding author.

## Footnotes

1
https://github.com/piloubazin/cerebellar-cortex
