# Supplementary material for: Neurocognition, cerebellar functions and psychiatric features in spinocerebellar ataxia type 34: a case series
Source: Front Comput Neurosci. 2025 Dec 9;19:1710961. doi: 10.3389/fncom.2025.1710961 (PMC12722519; doi:10.3389/fncom.2025.1710961)
Supplement: Supplementary file 1 [file Table_1.docx]

**Supplementary Table 1 – Neurocognitive and motor test battery**

This table summarizes the neurocognitive and motor tasks used in the study, grouped by the primary domains they assess to highlight their role in evaluating overall function.

| **Neurocognitive domain** | **Function** | **Test** |
| --- | --- | --- |
| **Verbal functions** | Verbal comprehension  Naming ability  Phonemic fluency  Semantic fluency | WAIS-IV – Vocabulary ^1^  Boston Naming Test ^2^  D-KEFS – Verbal Fluency Test, Letter Fluency ^3^  D-KEFS – Verbal Fluency Test, Category Fluency ^3^ |
| **Executive functions** | Inhibition  Shifting  Auditory working memory  Visuospatial working memory | D-KEFS – Color-Word Interference Test, Inhibition ^3^  D-KEFS – Trail Making Test, Number-Letter Switching ^3^  WAIS-IV – Digit Span, Sequencing ^1^  CANTAB – Spatial Span (*SSPFSL*) ^4^ |
| **Attention and**  **processing speed** | Visual scanning  Processing speed | D-KEFS – Trail Making Test, Visual Scanning ^3^  WAIS-IV – Symbol Search and Coding ^1^ |
| **Learning and memory** | Verbal learning  Verbal immediate recall  Verbal delayed recall  Verbal recognition  Visual learning  Visual immediate recall  Visual delayed recall  Visual recognition | RAVLT – Trials 1-5 ^5^  RAVLT – Trial 7  RAVLT – Trial 8  RAVLT – Trial 9  CANTAB – Pair Associated Learning (*PALTEA28*) ^4^    CANTAB – Pair Associated Learning (*PALFAMS28*)  CANTAB – Pattern Recognition Memory (*PRMPCI*)  CANTAB – Pattern Recognition Memory (*PRMPCD*) |
| **Visuospatial perception and visuospatial abilities** | Visuoconstructive function  Visuospatial abilities  Visuospatial perception | WAIS-IV – Block Design (*No time bonus*) ^1^  WMS-III – Visual Reproduction-II, Copy Task (*Accuracy*) ^6^  VOSP – Cube Analysis and Silhouettes ^7^ |
| **Social cognition** | Theory of mind | CANTAB – Emotion Recognition Task (*ERTTH*) ^4^ |
| **Motor skills** | **Function** | **Test** |
| **Cerebellar functions** | Sensorimotor synchronization  Visuospatial adaptation  Motor speed | Finger Tapping – Production Mean ^8^  Prism Adaptation – Prism Error Mean ^9^  D-KEFS – Trail Making Test, Motor Speed ^3^ |

***Note.*** *CANTAB* = Cambridge Neuropsychological Test Automated Battery; *D-KEFS* = Delis–Kaplan Executive Function System; *RAVLT* = Rey Auditory Verbal Learning Test; *WAIS-IV* = Wechsler Adult Intelligence Scale, Fourth Edition; *WMS-III* = Wechsler Memory Scale, Third Edition. Descriptions of the *CANTAB* variables are provided in the Methods section.

**References**

^1^ Wechsler, D. (2008). *WAIS-IV Administration and Scoring Manual*. Pearson.

^2^ Goodglass, H., Kaplan, E., & Weintraub, S. (1983). *Boston naming test*. Lea &  
  Febiger.

^3^ Delis, D. C., Kaplan, E., & Kramer, J. H. (2001). *Delis-Kaplan executive function system* (D-KEFS). The Psychological Corporation.

^4^ Cambridge Cognition. (2019). *Digital cognitive assessments*. Cambridge Cognition. https://cambridgecognition.com/digital-cognitive-assessments/

^5^ Schmidt, M. (1996). *Rey auditory verbal learning test* (pp. 1-125). Western  
   Psychological Services.

^6^ Wechsler, D. (1997). *WMS-III: Wechsler memory scale administration and scoring manual*.  
   Psychological Corporation. 
^7^ Warrington, E. K., & James, M. (1991). *The Visual Object and Space Perception Battery.* Thames Valley Test Company.

^8^ Gustafsson, P., Kjell, K., Cundari, M., Larsson, M., Edbladh, J., Madison, G., Kazakova, O., & Rasmussen, A. (2023). The ability to maintain rhythm is predictive of ADHD diagnosis and profile. *BMC psychiatry*, *23*(1), 920. https://doi.org/10.1186/s12888-023-05401-8

^9^ Cundari, M., Vestberg, S., Hansson, A., Kennberg, J., Gustafsson, P., & Rasmussen, A. (2025). Sensorimotor functions, visuospatial perception and visuospatial abilities in adult attention deficit hyperactivity disorder and autism spectrum disorder. *Journal of the International Neuropsychological Society: JINS*, 1–13. Advance online publication. https://doi.org/10.1017/S1355617725000189
