# Supplementary material for: Neurocognition, cerebellar functions and psychiatric features in spinocerebellar ataxia type 34: a case series
Source: Front Comput Neurosci. 2025 Dec 9;19:1710961. doi: 10.3389/fncom.2025.1710961 (PMC12722519; doi:10.3389/fncom.2025.1710961)
Supplement: Supplementary file 2 [file Table_2.docx]

**Supplementary Table 2 – Detailed neurocognitive assessments**

**Case Report and Test Summary of Neurocognitive Assessment of P1**

**Clinical report**

The patient is a 72-year-old male at the time of neurocognitive testing. He first experienced symptoms at age 50 and was diagnosed 19 years after symptom onset. His medical history includes a diagnosis of sleep apnea and a single episode of unprovoked seizure in 2019. The patient reports low, non-clinically significant levels of anxiety, depression, and insomnia. No other psychiatric conditions were identified during the evaluation. Comorbidities and current medications might have a minor influence on test performance (Amlodipine 10 mg once daily, Bisoprolol 12.5 mg once daily, Losartan 100 mg once daily).

**Neurological examination** findings revealed vertical and horizontal gaze-evoked nystagmus (GEN), low-gain and saccadic smooth pursuit, and hypometric saccades. The patient exhibited mild ataxia of gait, upper limbs, and lower limbs. Tendon reflexes were absent. No extrapyramidal signs were present. **Neuroradiological examination** reveals mild deep white matter changes (Fazekas grade 1), mild global cortical atrophy (GCA 1), and mild right medial temporal (MTA 1) and moderate left medial temporal (MTA 2) and mild parietal atrophy (PA 1). There is moderate bilateral cerebellar atrophy, including the vermis, as well as moderate atrophy of the middle and superior cerebellar peduncles. Mild pontine atrophy is also noted. No signs of lacunar infarctions, larger infarcts, or other focal lesions were detected. Overall, the findings are consistent with moderate generalized atrophy, more pronounced in the cerebellum, along with mild SVD.

**The patient’s estimated premorbid cognitive level** is in the high average range (≈1 SD above the mean), supported by 17 years of education, he completed Master’s-level studies. Results for **verbal functions** revealed mild deficits in verbal comprehension, semantic processing, and expressive language, while confrontational naming was slightly below premorbid level. Both phonemic and semantic verbal fluency were markedly reduced, characterized by a high frequency of set-loss errors, indicative of **executive dysfunction**, particularly in cognitive monitoring and set maintenance. Additionally, the patient demonstrated diminished performance in tasks assessing visuospatial and auditory working memory, as well as inhibitory control. In contrast, cognitive flexibility, as measured by set-shifting tasks, was within normative expectations. **Attention and processing speed** were also reduced. Lower scores emerge with **episodic memory** across both visual and verbal modalities, with deficits evident in learning, immediate recall, delayed recall, and most prominently in verbal recognition. Although the patient demonstrates adequate visual sensory function to support assessment, **visuospatial and visuoperceptual abilities** are significantly compromised. Notable deficits were observed in visuoconstructive problem-solving and overall spatial processing. **Social cognition** also appears to be affected, as reflected by difficulties in tasks involving emotion recognition.

Results for **motor abilities and cerebellar functions** show variability. The patient demonstrated slightly reduced motor speed and sensorimotor synchronization, along with severe impairments in motor learning and visuospatial adaptation. The severity of cerebellar ataxia has shown **slow progression**, with the SARA (Scale for the Assessment and Rating of Ataxia) score increasing from **7.5/40 (mild)** in June 2021 to **12.5/40 (moderate)** in July 2024 reflecting a **progression rate** of approximately **0.62%** per year.

**Table 1. Neurocognitive and Motor Test Performance Overview – P1.**

| **Neurocognitive domain** | **Test** | **RS** | **SS** | **Z** | **Notes** |
| --- | --- | --- | --- | --- | --- |
| **Verbal functions** | WAIS-IV – Vocabulary | 31 | 9 |  |  |
|  | D-KEFS – Letter Fluency | 36 | 10 |  | *Errors = 8* |
|  | D-KEFS – Category Fluency | 36 | 11 |  | *Errors = 1*  *Total Errors = 9 = 1 SS* |
|  | Boston Naming Test | 54 |  | 0.5 |  |
| **Executive functions** | WAIS-IV – Digit Span, Forward | 8 | 10 |  |  |
|  | WAIS-IV – Digit Span, Backward | 6 | 8 |  |  |
|  | WAIS-IV – Digit Span, Sequencing | 7 | 9 |  |  |
|  | WAIS-IV – Digit Span, Total | 21 | 9 |  |  |
|  | CANTAB – Spatial Span (*SSPFSL*) | 2 |  | -0.25 |  |
|  | D-KEFS – Trail Making Test, Number-Letter Switching | 93 | 12 |  |  |
|  | D-KEFS – Color-Word Interference Test, Inhibition | 76 | 10 |  |  |
| **Attention and**  **processing speed** | WAIS-IV – Symbol Search | 22 | 10 |  |  |
|  | WAIS-IV – Coding | 43 | 8 |  |  |
|  | D-KEFS – Trail Making Test, Visual Scanning | 32 | 9 |  |  |
| **Verbal episodic memory** | RAVLT – Total Learning (Trials 1–5) | 36 | 9 | -0.15 |  |
|  | RAVLT – B List | 3 | 8 | -0.56 |  |
|  | RAVLT – A List after Interference | 8 | 10 | -0.03 |  |
|  | RAVLT – A List after 30 Minutes | 9 | 13 | 0.83 |  |
|  | RAVLT – Recognition | 9 | 1 | -2.87 |  |
| **Visual episodic**  **memory** | CANTAB – PAL, Learning (*PALTEA28*) | 16 |  | -0.20 |  |
|  | CANTAB – PAL, Immediate Recall (*PALFAMS28*) | 12 |  | -0.15 |  |
|  | CANTAB – Pattern Recognition, Delayed (*PRMPCI*) | 77 |  | -0.74 |  |
|  | CANTAB – Pattern Recognition, Recognition (*PRMPCD*) | 61 |  | -0.92 |  |
| **Visuospatial perception**  **and abilities** | VOSP – Detection Screening | 20 | * |  |  |
|  | VOSP – Silhouettes | 19 | * |  |  |
|  | VOSP – Cube Analysis | 10 | * |  |  |
|  | WAIS-IV – Block Design (*No Time Bonus*) | 32 | 9 |  |  |
|  | WMS-III – Visual Reproduction-II, Copy Task | 84 | 5 |  |  |
| **Social cognition** | CANTAB – Emotion Recognition Task (*ERTTH*) | 26 | 7** |  |  |
| **Motor skills** |  |  |  |  |  |
| **Cerebellar functions** | Finger Tapping – Production Mean | 521 | 12** |  |  |
|  | Prisma Adaptation – Prism Error Mean | 5.9 | 1** |  |  |
|  | D-KEFS – Trail Making Test, Motor Speed | 40 | 11 |  |  |

***Note***. Raw Scores (RS): Untransformed test results, representing the number of items answered correctly, errors, or the total points earned before any comparison to norms. Scaled Scores (SS): Obtained after conversion from Raw Scores, they have a range from 1 to 19, with a mean (M) of 10 and a standard deviation (SD) of 3. Z-scores (Z): Mean (M) = 0, standard deviation (SD) = 1. *= These measures were not included in the analysis due to the lack of Swedish normative data, the results are reported for descriptive purposes. **=Scaled scores were calculated based on our sample data.

**Case Report and Test Summary of Neurocognitive Assessment of P2**

**Clinical report**

The patient is a 76-year-old male at the time of neurocognitive testing. He first experienced symptoms at age 41 and was diagnosed 32 years after symptom onset. He met diagnostic criteria for mild cognitive impairment in 2021 and suffered a Type 1 myocardial infarction in 2024. Additional comorbidities identified in the medical records include psoriasis, hypertension, sleep apnea, and atherosclerotic heart disease. The patient reports low, non-clinically significant levels of anxiety, depression, and insomnia. No other psychiatric conditions were identified during evaluation. Comorbidities and current medications partially influenced the test results (Atorvastatin 80 mg once daily, Candesartan 8 mg once daily, Citalopram 30 mg once daily, Clopidogrel 75 mg once daily, Dapagliflozin 10 mg once daily, Ezetimibe 10 mg once daily, Trombyl 75 mg once daily).

**Neurological examination** findings included dysarthria, horizontal and vertical GEN, hypometric saccades, low-gain and saccadic smooth pursuit; and an increased gain vestibulo-ocular reflex (VOR), gait ataxia, and ataxia in both upper and lower limbs. Additional signs were strabismus, unilateral ptosis, diplopia. No extrapyramidal signs were observed. Tendon reflexes were decreased. The patient repeatedly self-reported complex visual hallucinations, such as seeing a “threatening black cat” and experiencing a “feeling of being observed”. **Neuroradiological examination** reveals mild deep white matter changes (Fazekas grade 1), mild-to-moderate global cortical atrophy (GCA 1–2), and mild medial temporal (MTA 1) and parietal atrophy (PA 1). There is moderate bilateral cerebellar atrophy, including the vermis, as well as mild atrophy of the middle and moderate of the superior cerebellar peduncles. Mild pontine atrophy is also noted. No signs of lacunar infarctions, larger infarcts, or other focal lesions were detected. Overall, the findings are consistent with moderate generalized and cerebellar atrophy, accompanied by mild signs of SVD. Follow-up MRIs from 2023 and 2024 show no significant change.

**The patient’s estimated premorbid cognitive level** is in the high average range (≈1 SD above the mean), supported by 16 years of education, he completed Master’s-level studies. **Verbal functions** are strongly reduced. The results indicate impairments in verbal comprehension, semantic knowledge, verbal expression, and confrontational word retrieval. Both phonemic and semantic fluency are markedly impaired, with a high number of set-loss errors, suggesting executive dysfunctions such as difficulties with inhibition and short-term memory. **Executive functions** are clearly compromised. The patient showed impairments in visuospatial and auditory working memory, cognitive flexibility, and inhibitory control. **Attention and processing speed** were also robustly reduced. **Episodic memory** is impaired across both visual and verbal modalities, with impairments observed in learning, immediate recall, delayed recall, and recognition. Despite the patient demonstrates adequate visual sensory capacity to permit assessment of object and spatial perception, **visuospatial and visuoperception** are also decreased. However, deficits were identified in visuo-constructive problem solving, visuospatial discrimination, and recognition of objects and animals from unconventional perspectives. Severe impairments were noted on figure-copy tasks, which may be partially attributable to motor dysfunction. **Social cognition** appears to be affected, as indicated by deficits in emotion recognition tasks.

Results for **motor abilities and cerebellar functions** are very low. The patient demonstrated marked difficulties in motor speed, sensorimotor synchronization, motor learning, sensorimotor coordination, and visuospatial adaptation. **The severity of cerebellar ataxia is progressive.** The SARA score increased from **8/40 (mild impairment)** on 2019-12-20 to **17.25/40 (severe impairment)** on July 2024, reflecting a **progression rate of 0.59%** per year.

**Table 2. Neurocognitive and Motor Test Performance Overview – P2.**

| **Neurocognitive domain** | **Test** | **RS** | **SS** | **Z** | **Notes** |
| --- | --- | --- | --- | --- | --- |
| **Verbal functions** | WAIS-IV – Vocabulary | 11 | 4 |  |  |
|  | D-KEFS – Letter Fluency | 14 | 4 |  | *Errors = 4* |
|  | D-KEFS – Category Fluency | 5 | 1 |  | *Errors = 13*  *Total Errors = 17 = 1 SS* |
|  | Boston Naming Test | 50.5 |  | -0.5 |  |
| **Executive functions** | WAIS-IV – Digit Span, Forward | 6 | 6 |  |  |
|  | WAIS-IV – Digit Span, Backward | 4 | 5 |  |  |
|  | WAIS-IV – Digit Span, Sequencing | 1 | 2 |  |  |
|  | WAIS-IV – Digit Span, Total | 11 | 3 |  |  |
|  | CANTAB – Spatial Span (*SSPFSL*) | 2 | * | -2.33 |  |
|  | D-KEFS – Trail Making Test, Number-Letter Switching | 240 | 1 |  |  |
|  | D-KEFS – Color-Word Interference Test, Inhibition | 180 | 1 |  |  |
| **Attention and**  **processing speed** | WAIS-IV – Symbol Search | 4 | 2 |  |  |
|  | WAIS-IV – Coding | 17 | 4 |  |  |
|  | D-KEFS – Trail Making Test, Visual Scanning | 108 | 1 |  |  |
| **Verbal episodic memory** | RAVLT – Total Learning (Trials 1–5) | 11 | 1 | -3.48 |  |
|  | RAVLT – B List | 4 | 10 | 0.06 |  |
|  | RAVLT – A List after Interference | 2 | 4 | -2.03 |  |
|  | RAVLT – A List after 30 Minutes | 3 | 5 | -1.67 |  |
|  | RAVLT – Recognition | 9 | 1 | -2.87 | *Errors = 5, B List = 4* |
| **Visual episodic**  **memory** | CANTAB – PAL, Learning (*PALTEA28*) | 62 |  | -2.33 |  |
|  | CANTAB – PAL, Immediate Recall (*PALFAMS28*) | 3 |  | -2.05 |  |
|  | CANTAB – Pattern Recognition, Delayed (*PRMPCI*) | 77 |  | -0.74 |  |
|  | CANTAB – Pattern Recognition, Recognition (*PRMPCD*) | 38 |  | -2.05 |  |
| **Visuospatial perception**  **and abilities** | VOSP – Detection Screening | 16 | *** |  |  |
|  | VOSP – Silhouettes | 17 | *** |  |  |
|  | VOSP – Cube Analysis | 8 | *** |  |  |
|  | WAIS-IV – Block Design (*No Time Bonus*) | 16 | 6 |  |  |
|  | WMS-III – Visual Reproduction-II, Copy Task | 59 | 1 |  |  |
| **Social cognition** | CANTAB – Emotion Recognition Task (*ERTTH*) | 13 | 1** |  |  |
| **Motor skills** |  |  |  |  |  |
| **Cerebellar functions** | Finger Tapping – Production Mean | 445 | 5 ** |  |  |
|  | Prisma Adaptation – Prism Error Mean | 5.7 | 1 ** |  |  |
|  | D-KEFS – Trail Making Test, Motor Speed | 140 | 1 |  |  |

***Note***. Raw Scores (RS): Untransformed test results, representing the number of items answered correctly, errors, or the total points earned before any comparison to norms. Scaled Scores (SS): Obtained after conversion from Raw Scores, they have a range from 1 to 19, with a mean (M) of 10 and a standard deviation (SD) of 3. Z-scores (Z): Mean (M) = 0, standard deviation (SD) = 1. *= These measures were not included in the analysis due to the lack of Swedish normative data, the results are reported for descriptive purposes. **=Scaled scores were calculated based on our sample data.

**Case Report and Test Summary of Neurocognitive Assessment of P3**

**Clinical report**

The patient is a 76-year-old female at the time of neurocognitive testing. She first experienced symptoms at age 61 and was diagnosed 9 years after symptom onset. Her medical history includes the following comorbidities: epileptic seizures, psoriasis, constipation, mild cognitive impairment, and insomnia. She experienced a stroke or transient ischemic attack in 2020 and has a pacemaker, for this reason she was not able to be scanned with MRI. The patient reports clinically significant levels of mild anxiety and moderate insomnia. No other psychiatric conditions were identified during the evaluation. Comorbidities and current medications might have a minor influence on test performance (Metaprolol 100 mg once daily, Trombyl 160 mg once daily, Simvastatin 20 mg once daily).

**Neurological examination** findings revealed dysarthria, vertical and horizontal GEN, low-gain and saccadic smooth pursuit, and hypometric saccades. The patient exhibited ataxia of gait, upper limbs, and lower limbs. Tendon reflexes were normal. No extrapyramidal signs were present. **Neuroradiological examinations** reveal mild deep white matter changes (Fazekas grade 1), mild global cortical atrophy (GCA 1), mild medial temporal atrophy (MTA 1) and in the parietal region (PA 1). There is moderate bilateral cerebellar atrophy, along with moderate atrophy of the middle and superior cerebellar peduncles, and more pronounced atrophy of the vermis. Mild pontine atrophy is also observed. No evidence of lacunar infarctions, major infarcts, or other focal lesions was found. Overall, the findings are consistent with mild generalized atrophy and extensive cerebellar degeneration, including involvement of the middle and superior cerebellar peduncles, as well as widespread SVD.

**The patient’s premorbid cognitive level** was estimated near the average (≈0-0.5 SD above the mean). She completed gymnasium followed by two years of extra education. The **verbal functions** are decreased. Results show impairments for verbal comprehension, semantic knowledge, verbal expression and confrontational word retrieval. Phonemic fluency is also reduced. **Executive functions** partially are impaired. Results demonstrate decreased performances in visuospatial, but not in auditory working memory. Furthermore, flexibility and inhibitory control are heavily impaired. **Attention and processing speed** are also decreased. **Verbal episodic memory** shows more impairment than **visual episodic memory**. The results show impairments in learning, immediate recall, delayed recall and recognition. Even if the patient demonstrates sufficient visual sensory capacity allowing for testing of objects and space perception, **visuospatial perception and abilities** are impaired. Results show impairments for visuo-constructive problem solving, deficits in visuospatial perception, visual discrimination, and object and animal recognition from unconventional perspectives. Severe impairments were observed in the figure-copy tasks, which may partly be a result of motor impairment. **Social cognition** might be decreased. Results show severe impairments in the emotion recognition task.

The results for **motor abilities and cerebellar functions** are reduced. Results show decreased performance in motor speed. Sensorimotor synchronization is in line with premorbid estimated level. Motor learning, sensorimotor coordination and visuospatial adaptation are also severely impaired. The severity of cerebellar ataxia has shown **a slow progression**, with the SARA score increasing from **6.5/40 (mild)** in June 2021 to **21.5/40 (severe)** in July 2024 reflecting a **progression rate** of approximately **1.43%** per year.

**Table 3. Neurocognitive and Motor Test Performance Overview – P3.**

| **Neurocognitive domain** | **Test** | **RS** | **SS** | **Z** | **Notes** |
| --- | --- | --- | --- | --- | --- |
| **Verbal functions** | WAIS-IV – Vocabulary | 24 | 7 |  |  |
|  | D-KEFS – Letter Fluency | 20 | 6 |  | *Errors = 1* |
|  | D-KEFS – Category Fluency | 40 | 13 |  | *Errors = 1*  *Total Errors = 2 = 11 SS* |
|  | Boston Naming Test | 47 |  | -1.1 |  |
| **Executive functions** | WAIS-IV – Digit Span, Forward | 10 | 10 |  |  |
|  | WAIS-IV – Digit Span, Backward | 7 | 9 |  |  |
|  | WAIS-IV – Digit Span, Sequencing | 6 | 9 |  |  |
|  | WAIS-IV – Digit Span, Total | 23 | 10 |  |  |
|  | CANTAB – Spatial Span (*SSPFSL*) | 4 |  | *-*0.99 |  |
|  | D-KEFS – Trail Making Test, Number-Letter Switching | 240 | 1 |  |  |
|  | D-KEFS – Color-Word Interference Test, Inhibition | 102 | 5 |  |  |
| **Attention and**  **processing speed** | WAIS-IV – Symbol Search | 11 | 6 |  |  |
|  | WAIS-IV – Coding | 34 | 8 |  |  |
|  | D-KEFS – Trail Making Test, Visual Scanning | 38 | 7 |  | *Errors = 2 = 4% Cumulative Percentile* |
| **Verbal episodic memory** | RAVLT – Total Learning (Trials 1–5) | 27 | 6 | -1.35 |  |
|  | RAVLT – B List | 2 | 6 | -1.19 |  |
|  | RAVLT – A List after Interference | 6 | 8 | -0.70 |  |
|  | RAVLT – A List after 30 Minutes | 5 | 8 | -0.83 |  |
|  | RAVLT – Recognition | 11 | 5 | -1.53 | *Errors = 1, B List = 9* |
| **Visual episodic**  **memory** | CANTAB – PAL, Learning (*PALTEA28*) | 41 |  | -1.17 |  |
|  | CANTAB – PAL, Immediate Recall (*PALFAMS28*) | 9 |  | -0.55 |  |
|  | CANTAB – Pattern Recognition, Delayed (*PRMPCI*) | 94 |  | 0.28 |  |
|  | CANTAB – Pattern Recognition, Recognition (*PRMPCD*) | 72 |  | -0.44 |  |
| **Visuospatial perception**  **and abilities** | VOSP – Detection Screening | 20 | *** |  |  |
|  | VOSP – Silhouettes | 10 | *** |  |  |
|  | VOSP – Cube Analysis | 5 | *** |  |  |
|  | WAIS-IV – Block Design (*No Time Bonus*) | 20 | 6 |  |  |
|  | WMS-III – Visual Reproduction-II, Copy Task | 69 | 2 |  |  |
| **Social cognition** | CANTAB – Emotion Recognition Task (*ERTTH*) | 14 | 1** |  |  |
| **Motor skills** |  |  |  |  |  |
| **Cerebellar functions** | Finger Tapping – Production Mean | 503 | 10** |  |  |
|  | Prisma Adaptation – Prism Error Mean | 3.2 | 6** |  |  |
|  | D-KEFS – Trail Making Test, Motor Speed | 71 | 7 |  |  |

***Note***. Raw Scores (RS): Untransformed test results, representing the number of items answered correctly, errors, or the total points earned before any comparison to norms. Scaled Scores (SS): Obtained after conversion from Raw Scores, they have a range from 1 to 19, with a mean (M) of 10 and a standard deviation (SD) of 3. Z-scores (Z): Mean (M) = 0, standard deviation (SD) = 1. *= These measures were not included in the analysis due to the lack of Swedish normative data, the results are reported for descriptive purposes. **=Scaled scores were calculated based on our sample data.

**Case Report and Test Summary of Neurocognitive Assessment of P4**

**Clinical report**

The patient is a 52-year-old female at the time of neurocognitive testing. She first experienced symptoms at age 46 and was diagnosed 3 years after symptom onset. She has been diagnosed with schizophrenia since the age of 25 and has since experienced recurrent psychotic episodes, depressive symptoms, and delusional thinking. At the time of assessment, she reported clinically significant levels of anxiety. Insomnia and depressive symptoms were rated as low. No other psychiatric conditions emerged during the clinical evaluation. Comorbidities and current pharmacological treatment are likely to have influenced test performance (Lergigan 1 mg twice daily, Zyprexa 5 mg once daily).

**Neurological examination** findings revealed mild dysarthria, limited upgaze, horizontal GEN, ataxia of gait, upper limbs, and lower limbs. Tendon reflexes were normal. No extrapyramidal signs were present. **Neuroradiological examinations** reveals mild deep white matter changes (Fazekas grade 1), mild global cortical atrophy (GCA 1), mild medial temporal (MTA 1) and moderate parietal atrophy (PA 2). There is severe bilateral cerebellar atrophy, including the vermis, along with moderate atrophy of the middle cerebellar peduncles and severe atrophy of the superior cerebellar peduncles. Mild pontine atrophy is also noted. No signs of lacunar infarctions, larger infarcts, or other focal lesions were detected. Overall, the findings are consistent with an extensive cerebellar atrophy, including atrophy of the middle and superior cerebellar peduncles, along with mild small vessel disease (SVD).

The patient’s **premorbid cognitive** level was estimated to be low average (≈-1 SD below the mean). She completed high school, followed by one additional year of education. **Verbal functions** are decreased. Results show impairments for confrontational word retrieval. Results in phonemic and semantic fluency tasks show a high number of set-loss errors which indicate impairments in executive functions such as inhibition and short-term memory. However, the patient demonstrated reduced performance in visuospatial and auditory working memory, as well as in inhibitory control. Cognitive flexibility was generally within expected limits. **Attention and processing speed** were also significant reduced. **Visual and verbal episodic memory** are impaired. The results show impairments in learning, immediate recall, delayed recall and recognition. The patient demonstrates sufficient visual sensory capacity allowing for testing of objects and space perception. **Visuospatial perception and abilities** are decreased, with difficulties in visuo-constructive problem solving, deficits in visuospatial perception, visual discrimination, and object and animal recognition from unconventional perspectives. Severe impairments were observed in the figure-copy tasks, which may partly be a result of motor impairment. **Social cognition** might be decreased. Results show deficits in emotion recognition task.

The results for **motor abilities and cerebellar** **functions** are impaired. Results show strong impairment in motor speed. Deficits emerged in sensorimotor synchronization. Motor learning, sensorimotor coordination and visuospatial adaptation are also severely impaired.

The severity of cerebellar ataxia has shown **a slow progression**, with the SARA score increasing from **5/40 (mild)** in 2021 to **22/40 (moderate)** in October 2024 reflecting a **progression rate** of approximately **2.44%** per year.

**Table 4. Neurocognitive and Motor Test Performance Overview – P4.**

| **Neurocognitive domain** | **Test** | **RS** | **SS** | **Z** | **Notes** |
| --- | --- | --- | --- | --- | --- |
| **Verbal functions** | WAIS-IV – Vocabulary | 19 | 5 |  |  |
|  | D-KEFS – Letter Fluency | 21 | 5 |  | *Errors = 6* |
|  | D-KEFS – Category Fluency | 26 | 5 |  | *Errors = 9*  *Total Errors = 15 = 1 SS* |
|  | Boston Naming Test | 43 | 1 | -3 |  |
| **Executive functions** | WAIS-IV – Digit Span, Forward | 7 | 5 |  |  |
|  | WAIS-IV – Digit Span, Backward | 5 | 5 |  |  |
|  | WAIS-IV – Digit Span, Sequencing | 0 | 1 |  |  |
|  | WAIS-IV – Digit Span, Total | 12 | 4 |  |  |
|  | CANTAB – Spatial Span (*SSPFSL*) | 3 |  | -2.33 |  |
|  | D-KEFS – Trail Making Test, Number-Letter Switching | 240 | 1 |  |  |
|  | D-KEFS – Color-Word Interference Test, Inhibition | 98 | 3 |  |  |
| **Attention and**  **processing speed** | WAIS-IV – Symbol Search | 18 | 5 |  |  |
|  | WAIS-IV – Coding | 20 | 2 |  |  |
|  | D-KEFS – Trail Making Test, Visual Scanning | 44 | 2 |  |  |
| **Verbal episodic memory** | RAVLT – Total Learning (Trials 1–5) | 20 | 1 | -3.41 |  |
|  | RAVLT – B List | 1 | 4 | -2.14 |  |
|  | RAVLT – A List after Interference | 7 | 7 | -1.04 |  |
|  | RAVLT – A List after 30 Minutes | 3 | 3 | -2.23 |  |
|  | RAVLT – Recognition | 10 | 2 | -2.70 | *B List = 2* |
| **Visual episodic**  **memory** | CANTAB – PAL, Learning (*PALTEA28*) | 44 |  | -1.88 |  |
|  | CANTAB – PAL, Immediate Recall (*PALFAMS28*) | 5 |  | -2.05 |  |
|  | CANTAB – Pattern Recognition, Delayed (*PRMPCI*) | 44 |  | -2.33 |  |
|  | CANTAB – Pattern Recognition, Recognition (*PRMPCD*) | 38 |  | -2.05 |  |
| **Visuospatial perception**  **and abilities** | VOSP – Detection Screening | 19 | *** |  |  |
|  | VOSP – Silhouettes | 17 | *** |  |  |
|  | VOSP – Cube Analysis | 4 | *** |  |  |
|  | WAIS-IV – Block Design (*No Time Bonus*) | 16 | 2 |  |  |
|  | WMS-III – Visual Reproduction-II, Copy Task | 63 | 1 |  |  |
| **Social cognition** | CANTAB – Emotion Recognition Task (*ERTTH*) | 22 | 4** |  |  |
| **Motor skills** |  |  |  |  |  |
| **Cerebellar functions** | Finger Tapping – Production Mean | 348 | 1** |  |  |
|  | Prisma Adaptation – Prism Error Mean | 3.7 | 5** |  |  |
|  | D-KEFS – Trail Making Test, Motor Speed | 133 | 1 |  |  |

***Note***. Raw Scores (RS): Untransformed test results, representing the number of items answered correctly, errors, or the total points earned before any comparison to norms. Scaled Scores (SS): Obtained after conversion from Raw Scores, they have a range from 1 to 19, with a mean (M) of 10 and a standard deviation (SD) of 3. Z-scores (Z): Mean (M) = 0, standard deviation (SD) = 1. *= These measures were not included in the analysis due to the lack of Swedish normative data, the results are reported for descriptive purposes. **=Scaled scores were calculated based on our sample data.
