# Supplementary material for: Neurocognition, cerebellar functions and psychiatric features in spinocerebellar ataxia type 34: a case series
Source: Front Comput Neurosci. 2025 Dec 9;19:1710961. doi: 10.3389/fncom.2025.1710961 (PMC12722519; doi:10.3389/fncom.2025.1710961)
Supplement: Supplementary file 3 [file Table_3.DOCX]

**Supplementary Table 3 – Magnetic Resonance Imaging - Sequence parameters**

This table summarizes the magnetic resonance imaging sequences used in the study. All scans used the same B0 shim settings; 2^nd^ order image-based shimming.

|  | **MPRAGE** | **MP2RAGE Cerebellum** | **MP2RAGE whole brain** | **3D FLAIR** |
| --- | --- | --- | --- | --- |
| **Sequence type** | 3D IR FFE | 3D TFE dual TI IR | 3D TFE dual TI IR | 3D IR TSE |
| **Receive channels** | 40 | 40 | 40 | 40 |
| **Acquisition time (min:sec)** | 00:55 | 09:10 | 04:10 | 05:15 |
| **Orientation** | Sagittal | Axial | Sagittal | Sagittal |
| **Phase direction** | AP | AP | AP | AP |
| **FOV (mm**^3^**) (FH × AP × RL)** | 212×238×245 | 80×224×180 | 224×224×180 | 250×250×250 |
| **Acquisition matrix** | 212×238×245 | 159×445×360 | 280×282×225 | 252×250×250 |
| **Acquired resolution (mm^3^)** | 1.0×1.0×1.0 | 0.5×0.5×0.5 | 0.8×0.8×0.8 | 1.0×1.0×1.0 |
| **Reconstructed res. (mm^3^)** | 0.9×0.9×0.9 | 0.5×0.5×0.5 | 0.8×0.8×0.8 | 0.8×0.8×0.8 |
| **Acceleration factor CS-SENSE** | 6 | 2 | 4 | 8 |
| **TR (ms)** | 6.0 | 6.2 | 6.2 | 7500 |
| **TE (ms)** | 2.4 | 2.6 | 2.2 | 300 |
| **TI (ms)** | 1200 | TI_1_: 900  TI_2_: 2750 | TI_1_: 900  TI_2_: 2750 | 2200 |
| **Cycle duration (ms)** | - | 5000 | 5000 | - |
| **Excitation flip angle (°)** | 7 | α_1_: 5  α_2_: 3 | α_1_: 5  α_2_: 3 | 60 |
| **Refocusing angle (°)** | - | - | - | 44 |
| **Echo train length** | 385 | 256 | 256 | 128 |
| **Echo spacing (ms)** | - | - | - | 4.1 |
| **Startup echoes** | - | - | - | 8 |
| **Bandwidth per pixel (Hz)** | 289 | 457 | 437 | 689 |
| **Fat suppression** | No | No | No | SPAIR |
| **T2prep pulse** | No | No | No | 4 pulses, TE=120 ms |
| **Tx phase settings** | Quadrature | Preset posterior brain | Quadrature | Quadrature |

***Note.*** *MP2RAGE* = Magnetization Prepared 2 Rapid Acquisition Gradient Echo; *MPRAGE* = Magnetization Prepared Rapid Acquisition Gradient Echo; *FLAIR* = Fluid-Attenuated Inversion Recovery; *TFE* = Turbo Field Echo; *IR* = Inversion Recovery; *FFE* = Fast Field Echo; *TSE* = Turbo Spin Echo; *Ch* = receive channels; *TA* = acquisition time; *Ori*. = orientation; *Ph dir.* = phase direction; *FOV* = field of view (FH = foot–head; AP = anterior–posterior; RL = right–left); *Res.* = resolution; *Rec. res.* = reconstructed resolution; *CS-SENSE* = Compressed SENSE acceleration; *TR* = repetition time; *TE* = echo time; *TI* = inversion time; *α* = excitation flip angle; *ETL* = echo train length; *BW/px* = bandwidth per pixel; *SPAIR* = spectral attenuated inversion recovery; *T2prep* = T2-preparation pulse; *Tx* = transmit.

**Magnetic Resonance Imaging examination protocol**

After acquiring a survey and an 3D MPRAGE with 1 mm isotropic resolution, 2^nd^ order B0 shimming was executed in order to optimize the magnetic field homogeneity and minimize image distortions. The MPRAGE sequence was used for planning of the subsequent imaging of the cerebellum. These initial scans took approximately 5 minutes. This was followed by a high-resolution 3D MP2RAGE (Marques et al., 2010), 0.5 mm isotropic resolution, allowing for highly detailed, T1-weighted images of the cerebellum, providing a basis for subsequent tissue segmentation and cortical thickness estimation. The axial field of view (FOV) for this scan was placed parallel to the brainstem, focused on the cerebellum, to minimize signal loss from inflowing arterial blood and improve visualization of cerebellar vasculature (Priovoulos and Bazin, 2023). Accelerating the acquisition using a compressed SENSE factor (CS-SENSE) of 2, the sequence took 09:10 minutes. To mitigate the B1-losses that are commonly seen in the cerebellum at 7T, a tailored fixed phase setting of the 8 Tx-channels was used for this scan. This B1-distribution was verified using a DREAM B1-mapping technique (one 11 s scan) (Nehrke et al., 2014). Subsequently, an additional 3D MP2RAGE sequence with 0.8 mm isotropic resolution and CS-SENSE acceleration by a factor of 4 was acquired over of the whole brain (04:10 min). Finally, a DREAM B1-map using the default phase settings (11 s) and a 3D FLAIR (05:15 min) were acquired. Apart from spatial resolution, CS-SENSE factors and B1-phase settings, the MP2RAGE parameters were identical for the cerebellar and whole brain scans; inversion times TI_1_ = 913 ms and TI_2_ = 2750 ms, flip angles α_1_ = 5° and α_2_ = 3°, an inversion repetition time (cycle duration) of 5000 ms, excitation repetition time (TR) of 6.2 ms, 256 excitations. The magnitude and phase images for both inversion times were exported for offline processing, further described below. For the radiological review, the MP2RAGE UNI images were reconstructed from the two inversion times on the scanner.

**Magnetic Resonance Image analysis**

The cerebellar-focused MP2RAGEs were processed using the Nighres toolbox (Huntenburg et al., 2018), which is optimised for ultra-high-resolution 7T images. The images were denoised and segmented following Priovoulos & Bazin’s (2023) pipeline (<https://github.com/piloubazin/cerebellar-cortex>). To prepare the cerebellar MP2RAGE data for segmentation, magnitude and phase images acquired at both inversion times were denoised using a local complex principal components analysis (LCPCA) algorithm implemented in Nighres v1.4.0 (Huntenburg et al., 2018). This takes advantage of both magnitude and phase information to improve noise removal, while preserving fine anatomical details critical for downstream segmentation. Denoising was carried out using a local neighbourhood size of 2 voxels and a standard deviation cutoff of 1.10. Using Nighres’ mp2rage_t1_mapping() function, following denoising, quantitative T1 maps and UNI images (bias-field-corrected uniform T1w) were reconstructed (Huntenburg et al., 2018).  As to enable anatomically precise segmentation and alignment with standard atlases, the MP2RAGE slab images were spatially coregistered and normalised to MNI space (MNI 2009c) using a multistep registration pipeline implemented in Nighres(Huntenburg et al., 2018; Priovoulos and Bazin, 2023). All registrations used the embedded_antyspy() function, a wrapper around the Advanced Normalization Tools’ ANT v2.1.1 (Avants et al., 2009) registration methods. Using whole-head   UNI and INV2images, the cerebellar-focused images were first aligned to the full brain and then coregistered and fine-tuned to MNI152 nonlinear asymmetric template using SyN models (Mazziotta, 2001). Inverse transforms were applied to warp a cerebellum-specific binary mask into subject space, providing a precise outline of the cerebellum. Moreover, a cerebellar atlas outlining the different anatomical regions was warped into subject space (Diedrichsen et al., 2009). Non-cerebellar brain parts faded out using a soft tissue mask. Using this subject-native mask, cerebellar regions of interest (ROIs) were defined.

Tissue classification within the cerebellar region was performed using a multi-step segmentation pipeline implanted in the Nighres toolbox (Huntenburg et al., 2018; Priovoulos and Bazin, 2023) . By applying intensity-based fuzzy clustering, ridge-based structure enhancement, and anatomical priors, this approach delineates cerebellar grey matter (GM), white matter (WM), and cerebrospinal fluid (CSF). This approach generates probability maps across the different tissue classes and provides the advantage that each voxel can belong partly to more than one tissue type. As to not lose fine structures, recursive 2D ridge filtering was applied to the T1 maps, to enhance delineation of WM and CSF. Furthermore, to avoid the dura being misregistered as WM, it was explicitly modelled using a prior probability map. A final probability map was computed: WM was computed as the sum of the clustering results and ridge-enhanced WM maps minus the dura region. CSF as the FCM-derived clustering results and CSF ridge-enhanced map as well as the dura regions. After that, GM was computed as the complement to WM and CSF (GM = 1 – WM – CSF). The results were fine-tuned to match the cerebellar area and adjusted so each voxel had a clear probability between 0 and 1 respective to its tissue properties. In an additional step, the topology was corrected by focusing and refining WM as to later accurately measure the thickness of the cerebellar cortex.

Following tissue classification, Nighres’ Cortical Reconstruction Using Implicit Surface Evolution algorithm CRUISE (Huntenburg et al., 2018) was applied, using the three previously provided three probabilistic tissue priors (WM, GM, CSF). CRUISE allows for building a 3D model of the cerebellar cortex, enabling analysis of its shape and thickness and performs best for approximating cerebellar cortical surface (Priovoulos and Bazin, 2023). The algorithm uses a deformable model constrained by tissue priors and anatomical boundaries to compute three surface level sets: the grey-white boundary (GWB), the grey-CSF boundary (CGB), and the mid-GM surface (AVG). The level sets were converted into triangular surface meshes using the levelset_to_mesh() function in Nighres. The inner (GWB), outer (CGB), and average (AVG) surfaces were used in downstream analyses. Cortical thickness was calculated as the distance between GWB and CGB at every voxel.

The lobules of interest were analysed as ROIs, following the previously delineated steps, providing measures of cerebellar cortical thickness for each ROI in millimeter by reading per-vertex label and thickness scalars and matching labels to region names. Regional cortical thickness was calculated by averaging the thickness values within each functional ROI, allowing assessment of thickness variation across functional parcellations of the cerebellum. Cortical thickness was selected due to its association with granule layer density, which is decreased in individuals suffering from ataxia (Pascual-Castroviejo et al., 1994). The cerebellar cortical thickness values were compared between the two scanned individuals and control.

**References**

Avants, B., Tustison, N. J., and Song, G. (2009). Advanced Normalization Tools: V1.0. *Insight J.* doi: 10.54294/uvnhin

Diedrichsen, J., Balsters, J. H., Flavell, J., Cussans, E., and Ramnani, N. (2009). A probabilistic MR atlas of the human cerebellum. *Neuroimage* 47, S122. doi: 10.1016/s1053-8119(09)71166-8

Huntenburg, J. M., Steele, C. J., and Bazin, P.-L. (2018). Nighres: processing tools for high-resolution neuroimaging. *Gigascience* 7. doi: 10.1093/gigascience/giy082

Marques, J. P., Kober, T., Krueger, G., Van Der Zwaag, W., Van De Moortele, P.-F., and Gruetter, R. (2010). *MP2RAGE, a Self Bias-Field Corrected Sequence for Improved Segmentation and T-1-Mapping at High Field*. doi: 10.1016/j.neuroimage.2009.10.002

Mazziotta, J. C. (2001). Brain mapping: its use in patients with neurological disorders. *Rev. Neurol. (Paris)* 157, 863–871. Available at: https://www.ncbi.nlm.nih.gov/pubmed/11677410

Nehrke, K., Versluis, M. J., Webb, A., and Bornert, P. (2014). Volumetric B-1(+) Mapping of the Brain at 7T Using DREAM. *Magn. Reson. Med* 71. doi: 10.1002/mrm.24667

Pascual-Castroviejo, I., Gutierrez, M., Martinez Bermejo, A., Morales, C., and Hernandez Moneo, J. L. (1994). Does norman syndrome begin by primary degeneration of granular layer of cerebellum? *Pediatr. Neurol.* 11, 108. doi: 10.1016/0887-8994(94)90232-1

Priovoulos, N., and Bazin, P.-L. (2023). Methods for cerebellar imaging analysis. *Curr. Opin. Behav. Sci.* 54, 101328. doi: 10.1016/j.cobeha.2023.101328
